# The relationship between growth and pattern formation

**DOI:** 10.1002/reg2.55

**Published:** 2016-04-28

**Authors:** Susan V. Bryant, David M. Gardiner

**Affiliations:** ^1^ Department of Developmental and Cell Biology; ^2^ University of California IrvineIrvineCalifornia

**Keywords:** Growth, morphogen, pattern formation, positional information, regeneration

## Abstract

Successful development depends on the creation of spatial gradients of transcription factors within developing fields, and images of graded distributions of gene products populate the pages of developmental biology journals. Therefore the challenge is to understand how the graded levels of intracellular transcription factors are generated across fields of cells. We propose that transcription factor gradients are generated as a result of an underlying gradient of cell cycle lengths. Very long cell cycles will permit accumulation of a high level of a gene product encoded by a large transcription unit, whereas shorter cell cycles will permit progressively fewer transcripts to be completed due to gating of transcription by the cell cycle. We also propose that the gradients of cell cycle lengths are generated by gradients of extracellular morphogens/growth factors. The model of cell cycle gated transcriptional regulation brings focus back to the functional role of morphogens as cell cycle regulators, and proposes a specific and testable mechanism by which morphogens, in their roles as growth factors (how they were originally discovered), also determine cell fate.

## Introduction

The emergence, over developmental time, of the pattern and form of the embryo from the fertilized egg is a topic that has engaged the interest of biologists from the earliest days of modern embryology (Thompson 1917) to the present (Towers et al. 2012; Gilbert 2013). It is evident that this process involves both growth and pattern formation, but the functional relationship between these processes is as yet unclear. Over the last few decades, information about the timing of appearance of gene products, and the localization of molecules such as cytoplasmic determinants, signaling molecules, differentiation products, gene activators and repressors, as well as all the intermediate molecules needed to generate them and to ensure successful progression through the steps of development, has increased exponentially. This information rightfully is seen as crucial to understanding the process of development. However, diagrams of gene regulatory networks (GRNs) (Levine & Davidson 2005) are only one part of the equation. The increasing level of sophistication in the analysis of these networks and of the interactions between molecules necessary for their activities has created a sense of security that we are in fact learning more about how development functions. However, it is cells, not genes or molecules, that are the units of development. Ultimately, we need to understand how cells use gene products to work together to create an embryo, or to regenerate a leg, in order to track backwards to understand the role that specific molecules and genes play in enabling the relevant cellular behaviors.

The successful outcome of both development and regeneration is dependent on growth resulting from cellular proliferation, and on pattern formation leading to differentiation of cells in the right place at the right time. Much of the focus of research to discover and characterize GRNs has been on one or other of these processes. In the case of cell proliferation, a major focus is on what happens when the cell cycle becomes dysregulated, leading to cancer, despite the fact that tumor cells have many characteristics associated with undifferentiated stem cells and/or regeneration‐competent progenitor cells. In addition, GRNs identified from developing systems tend to focus on the regulation of cell fate, associated with concepts such as “specification,” “determination,” and “differentiation,” even though cells of the embryo are proliferating and giving rise to multiple generations of progeny with diverse fates. Since both of these processes (cellular proliferation and pattern formation) are occurring simultaneously during both embryonic development and regeneration, we consider it reasonable to pose the question as to whether or not there is a functional relationship between regulation of the cell cycle and regulation of cell fate. In this essay, we draw from studies of both regeneration and embryonic development to propose a mechanistic view of how growth and pattern formation are regulated coordinately.

## The linkage between growth and pattern formation

Regenerating animals provide unique insights into the organizational logic and pattern formation characteristics of cells and organs because, during regeneration, specific parts of developmental programs can be reactivated in response to injury. Embryonic development is initiated by fertilization of the egg and cell division continues in an uninterrupted fashion leading to all the diverse differentiated cells of the post‐embryonic body plan. In contrast, regeneration begins when injury triggers a cascade of events leading to the local activation of proliferation by quiescent progenitor cells, and only the missing parts of the pattern are reformed. Thus, by studying regeneration, it is possible to test for the signals that activate and recruit regeneration‐competent cells, as well as those that orchestrate the behavior of those cells to make the new pattern. Since the multiple tissues of the regenerating structure (e.g., a limb) are already present at the time of injury, it is possible to identify the progenitor cells for each of these tissues, as well as to identify those cells that function to control formation of the pattern of new structures that are regenerated. Hence regeneration provides the opportunity to do experiments to test for a relationship between growth and pattern formation.

Many experiments in animals capable of regeneration have involved grafting tissues from one location to another. Typically growth is induced at the graft−host junction, and extra (supernumerary) pattern is generated (see French et al. 1976; Bryant et al. 1981). The amount of extra growth and the resultant supernumerary pattern are predicted based on the differences in positional information (positional disparity) between the graft and host cells, such that growth continues until all positional disparities have been resolved. This process of “intercalation” has been demonstrated repeatedly in many models of regeneration, ranging from planaria to salamander limbs (see Bryant et al. 2002; Agata et al. 2003). The cell−cell interactions that mediate intercalation are based on location‐specific differences that exist between cells from different positions in the limb (positional information). Thus an intercalary response arises as a result of the interaction between pattern‐forming cells with dissimilar positional information. This interaction stimulates proliferation and pattern formation to resolve positional disparities or to regenerate the missing structures (French et al. 1976; Bryant et al., 1981 2002). In contrast, cells that do not induce intercalation by definition lack positional information, and they are instead responsive to pattern‐forming cells and have been described as pattern‐following cells (McCusker & Gardiner 2013, 2014; McCusker et al. 2015).

**Figure 1:**
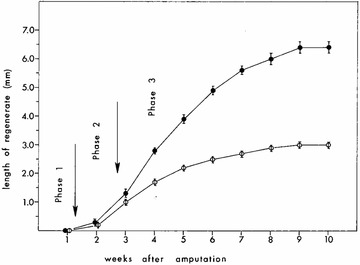
Both the amount and rate of growth during appendage regeneration are variable and are proportional to the amount of tissue that is removed by amputation. The lower curve shows growth over time from a distal amputation (less tissue removed) and the upper curve from a proximal amputation (more tissue removed). Growth rate is proportional to the disparity created such that regeneration is complete at the same time regardless of the amount to be replaced. From Iten and Bryant (1973).

As demonstrated, intercalation replaces only the part of the pattern that is missing, and therefore the amount of induced growth and pattern formation is directly proportional to the size of the disparity that is created. Less appreciated is the observation that the rate of growth is also a function of the size of the disparity (Fig. 1; Iten & Bryant 1973). As demonstrated experimentally by amputations at different levels along the proximal−distal axis (limb) as well as the rostral−caudal axis (tail), the time needed for complete regeneration of an appendage is the same regardless of the amount of tissue that needs to be replaced (Iten & Bryant 1973, 1976; Stocum 1980; Vincent et al. 2015). It appears that the early stages of blastema formation progress at equivalent rates; however, during the period of rapid growth, blastemas at more proximal levels increase in size at a more rapid rate than those from distal level amputations (Fig. 1). In the end, more tissue and more new pattern are formed from proximal level amputations than from distal level amputations, and thus the rate of regeneration is proportional to the amount of tissue to be replaced. Although the mechanism underlying variable rates of growth and pattern formation is as yet unknown, we presume it is related to the probability of whether a given cell−cell interaction will stimulate proliferation. Cells on either side of large disparities will need to divide more times to eliminate the disparity than will cells on either side of a small disparity. With smaller initial disparities, or in the later stages of resolving large disparities, positional differences between neighbors will be fewer, and thus the probability that interactions between neighboring cells having different positional information will become progressively lower. Consistent with this predictionbased on the mechanism of intercalation (French et al. 1976; Bryant et al., 1981, 2002), recent data on the growth fraction suggest that the differential rates of tail regeneration (proximal vs. distal amputations; Iten & Bryant 1976) are a consequence of changes in the proportion of cells in the blastema that are progressing through the cell cycle (Vincent et al. 2015). More detailed analyses of cell cycle kinetics at higher spatial and temporal resolution are needed to verify this relationship.

**Figure 2:**
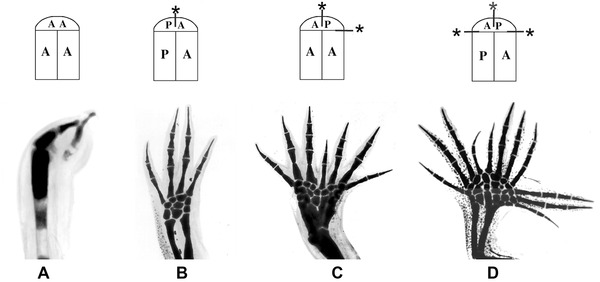
The relationship between the extent of the disparity and the amount of new pattern generated. The top row of the diagram illustrates the limb types generated by grafts of half limbs and early blastemas, and on the bottom row are the resulting limbs that have been stained to show the skeletal patterns. Positional confrontations between anterior (A) and posterior (P) cells are marked by an asterisk. Modified from Gardiner and Bryant (1998).

In addition to stimulating supernumerary growth and pattern formation, positional interactions between cells can also inhibit growth and pattern formation (Fig. 2). When normal interactions occur in response to limb amputation (Fig. 2B), cells from different positions around the limb circumference migrate toward the center of the wound (Gardiner et al. 1986; Endo et al. 2004) and interact to stimulate intercalation and regeneration of the relevant missing limb pattern dependent on the level of amputation (Bryant et al. 2002; McCusker et al. 2015). Supernumerary limb pattern is induced when the orientation of the blastema is altered relative to the stump either by rotating the blastema 180° or by grafting between left and right limbs so as to reverse the anterior−posterior (or dorsal−ventral) positional interactions (Fig. 2D; French et al. 1976; Bryant et al. 1981). In this situation additional limbs are induced to form at locations where anterior−posterior positional disparities are created (indicated by asterisks). In contrast, if a limb that lacks positional diversity is created surgically by replacing the posterior tissues to create a double‐anterior limb (Fig. 2A), both growth and pattern formation fail to occur and regeneration fails. An intermediate degree of growth and pattern formation is induced when a normal blastema (with both anterior and posterior information) is grafted to a double‐anterior stump (Fig. 2C). Thus the amount and pattern of growth are dependent on intercalary interactions between cells with positional information and are not influenced by systemic factors in the animal (Muneoka et al. 1989).

**Figure 3:**
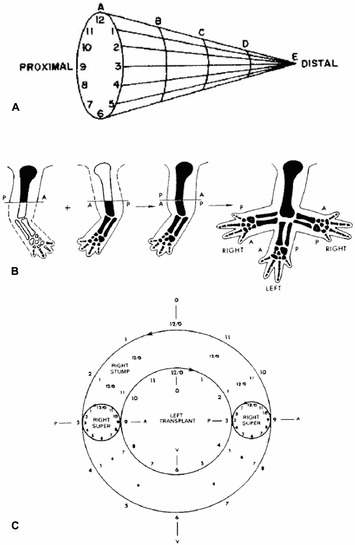
The polar coordinate model. As proposed for pattern formation in appendages, cells with positional information are arrayed in a two‐dimensional sheet under the epidermis and around internal structures (e.g., muscles, bones, nerves, and blood vessels). (A) The position identity of a cell is specified in relation to the limb circumference (1−12) and to the long axis of the appendage (A−E). (B) When limbs are grafted from left to right (and vice versa), cells with different positional identities are brought together at the graft−host interface, and intercalation is stimulated. (C) The host (outer circle) and grafted (inner circle) limbs are represented in this diagram indicating that the anterior−posterior axes (positions 9 and 3) have been reversed. Intercalation is stimulated at these positions, resulting in two supernumerary limbs (smaller circles), as illustrated also in (B). Since no positional disparities are created at the dorsal (12) or ventral (6) positions, no intercalation occurs and no supernumerary limbs form at those positions. Modified from Bryant et al. (1981) and French et al. (1976).

In the 1970s, it first became evident that growth and pattern formation were functionally linked during regeneration in animals as diverse as insects (French et al. 1976; Bryant et al. 1981) and amphibians (Bryant & Iten 1977, 1976). This remarkable conservation of the response to injury leading to regeneration led to the development of the polar coordinate model (PCM) to account in a formal way for the spectrum of results coming from a variety of grafting and regeneration experiments across species. The PCM (French et al. 1976; Bryant et al. 1981) proposes that cells in appendages have positional information about their location along the proximal−distal axis and around the circumference (Fig. 3A). As discussed above, when cells interact with non‐normal neighbors (cells with different positional information), such as after grafting or during healing following amputation, these positional disparities stimulate growth, leading to the intercalation of cells with intermediate positional values, thereby eliminating discontinuities in the pattern by generating supernumerary limbs with patterns and polarity that are predicted by the PCM (Fig. 3B, C). Intercalation continues until all disparities have been resolved. These studies opened a window into the relationship between growth and pattern because it is clear from them that positional disparities stimulate growth, and conversely that there is no growth without positional disparities. The PCM continues to be a useful formal model by which to understand experimental data (e.g., McCusker et al. 2014), and it can guide efforts aimed at understanding the nature of positional information in mammals (Phan et al. 2015).

## The role of digits in terminating limb growth and pattern

Although most research efforts have focused on how to stimulate growth and regeneration, there is the reciprocal and equally important challenge of how to stop these processes when the pattern has been restored. As noted above, the PCM predicts that growth and pattern formation will cease when all the positional disparities have been resolved (French et al. 1976; Bryant et al. 1981). Evidence that intercalation functions to terminate growth and pattern formation during both limb development and regeneration comes from experiments in which limbs are amputated at the level of the proximal base of the digits (Stock & Bryant 1981).

**Figure 4:**
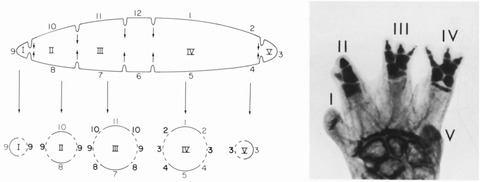
Termination of growth by symmetry of positional information. Symmetrical digit bases are generated when the more proximal limb circumference breaks up into multiple separate digit bases, which are self‐terminating (left diagram). Amputation at the digit bases leads to abnormal digit patterns during regeneration (right image). Roman numerals identify each digit from anterior (I) to posterior (V). Modified from Stock and Bryant (1981).

Amputation of a salamander limb at any level, except that of the digit bases, leads to restoration of exactly what was removed, including a normal pattern of digits. Amputation at the level of the digit bases produces a variety of abnormal patterns, including missing and extra digits (Stock & Bryant 1981) (Fig. 4). These results led to the recognition that during development and regeneration the asymmetrical circumference of the more proximal upper and lower arm segments becomes fragmented distally to form the digit bases, leading to formation of bilaterally symmetrical digits which are the terminating structures of the limb (Stock & Bryant 1981; Gardiner & Bryant 1989). Symmetrical structures, as illustrated in Figure 3, either occur naturally, such as digits (Fig. 4, Stock & Bryant 1981) and tails (Iten & Bryant 1976), or can be created experimentally in limbs by grafting to create symmetrical limb stumps, for example double‐anterior limbs (Fig. 2A, Bryant et al. 1982; Wigmore & Holder 1985; Wigmore 1986). Symmetrical structures are self‐terminating in that they progressively taper distally and eventually stop growing as a result of the progressive loss of positional information along the line of symmetry (Bryant et al. 1981). One exception to this is surgically created double‐symmetrical limbs in which each half has more than half of the positional information around the circumference, for example double‐posterior limbs (Bryant et al. 1982; Wigmore & Holder 1985; Wigmore 1986). These surgically created limbs are able to intercalate between the two halves to form supernumerary limbs rather than self‐terminating digit‐like structures. Similarly, during healing of wounds created by amputation at the level of the digit bases, the normally orderly fragmentation of the limb circumference into a series of symmetrical digit bases is disrupted, leading to a complex array of patterns including extra, fused and missing digits, all of which are consistent with having created positional discontinuities that are resolved subsequently by intercalation (Stock & Bryant 1981).

## The positional information grid

The critical role of positional information in the control of growth and pattern formation leads us to focus our attention on the question of what positional information is mechanistically, and how it is encoded in the tissue. Although the answers to these questions probably will not become known for some time, at this point we consider it reasonable to start with the premise that positional information is a cell‐based property, and thus there is a cell type in the body that creates and encodes positional information. This cell type established the pattern of positional information during development of the organ in the embryo (e.g., a limb), and this information can be accessed at a later time (as long as years later since old salamanders retain the ability to regenerate as they age). Since a regenerated limb also can regenerate when amputated, we presume that the positional information cells persist in the adult, and they are also regenerated when the limb regenerates. An immediate challenge is to identify and understand the biology of the positional information cells.

An important advantage of studying the cellular basis of positional information in a regenerating model system is that it is possible to identify and isolate specific cell types from the various tissues and then assay for the presence or absence of positional information. As discussed above, intercalation is stimulated when cells with different positional information interact, and supernumerary pattern is formed (Bryant et al. 2002; McCusker & Gardiner 2014). Thus assays for positional information typically involve grafting of cells to new locations and assaying for the formation of supernumerary structures. From such studies, it has been determined that cells in the connective tissues (generally referred to as fibroblasts) are the cells of the limb with positional information. These cells are distributed throughout the dermis, as well as in the connective tissue sheaths that surround blood vessels, muscles, bones, and nerves, thus creating a two‐dimensional grid of information adjacent to and surrounding all tissues (Lheureux 1983; Muneoka et al. 1986; Gardiner & Bryant 1989; Bryant et al. 2002; McCusker et al. 2015).

**Figure 5:**
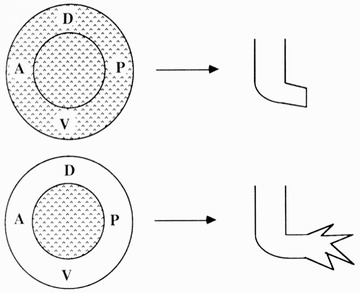
Positional information is localized in the dermis of the skin. Top: Complete irradiation of the limb inhibits regeneration after amputation. Bottom: Replacement of the irradiated skin with a cuff of unirradiated skin is sufficient to restore regenerative ability to the irradiated limb. Stippling indicates the tissues that have been X‐irradiated (A, anterior; D, dorsal; P, posterior; V, ventral). Based on Lheureux (1983).

The presence of positional cells in the dermis of the skin was demonstrated elegantly by Lheureux (Fig. 5; Lheureux 1983) who discovered that limb regeneration in urodeles occurs on an X‐irradiated limb stump that has been provided with a complete cuff of skin (dermis and epidermis) from an unirradiated donor. In the absence of grafted, unirradiated skin, an irradiated limb fails to regenerate when amputated. When rescued by dermal cells from the unirradiated, grafted dermis (grafted epidermal cells do not participate in rescuing regeneration), the regenerated limb forms from donor connective tissue cells and forms a normal pattern of structures (dermis, loose connective tissues, muscle fascia, skeleton, ligaments, and tendons) (Fig. 5). These results, as well as those from other grafting experiments, demonstrate that cells of the connective tissue have both the information to reform the entire limb pattern and the developmental potential to regenerate the tissues of the connective tissue lineage (Lheureux 1983; Rollman‐Dinsmore & Bryant 1984; Muneoka et al. 1986; Kragl et al. 2009). In addition to grafts of an entire circumference of skin, such as in the Lheureux experiments, even small skin grafts induce formation of supernumerary limb pattern, so long as the host site is sufficiently different from that of the donor site in terms of positional information (typically the host and graft sites are on opposite sides of the limb, e.g., anterior into posterior, dorsal into ventral, and vice versa) (Rollman‐Dinsmore & Bryant 1984).

**Figure 6:**
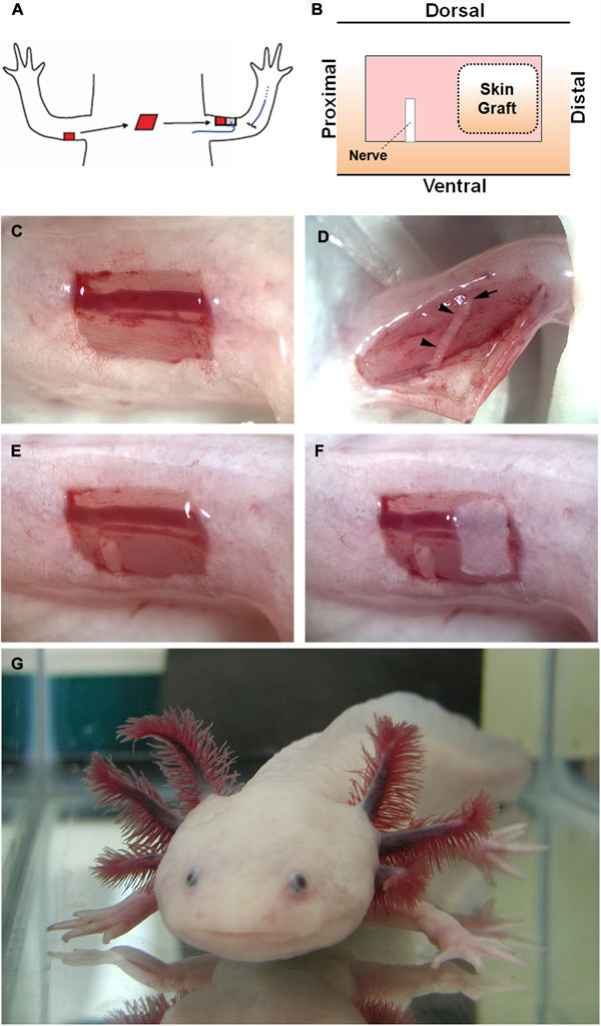
Making an accessory axolotl limb (ALM; Endo et al. 2004). (A) Ectopic limbs with normal pattern can be induced to form in response to a wound to which a nerve (blue) is surgically deviated, and a piece of skin (red) from the opposite side of the limb (posterior) is grafted into the host site (anterior). (B) Illustration of the final arrangement of wound, deviated nerve, and skin graft. (C)−(F) Images of the surgical steps in the ALM beginning with the full‐thickness skin wound (C), the exposed brachial nerve prior to being surgically deviated (D), the deviated nerve within the wound bed (E), and the grafted posterior skin in combination with the deviated nerve (F, as illustrated in B). This is sufficient to generate a supernumerary limb as shown in (G) (two left arms). Modified from Lee et al. (2013).

The ability of small grafts of connective tissue cells to induce supernumerary limb pattern led to the development of a simplified experimental model of regeneration, the accessory limb model (ALM) (Fig. 6; Endo et al. 2004; Satoh et al. 2007). This is a gain‐of‐function assay for signaling that induces blastema formation as well as for signaling associated with positional information. In the ALM, a full‐thickness skin wound is created on one side of the limb (typically on the anterior) (Fig. 6C), the brachial nerve is surgically deviated to the wound site (Fig. 6D, E), and a piece of skin from the opposite side of the limb (posterior) is grafted into the wound bed (Fig. 6F). In response, a well‐patterned ectopic limb is induced to form at high frequency (Fig. 6G). The ALM thus demonstrates the three essential requirements for limb regeneration: a wound, nerve signals, and disparity of positional information. The ALM has led in recent years to the demonstration that a cocktail of mammalian growth factors (bone morphogenetic protein [BMP] and fibroblast growth factor [FGF]) cansubstitute for the nerve signals necessary to induce blastema formation (Makanae et al. 2013, 2014; Satoh et al. 2015). Similarly, this assay has led to the discovery that positional information is encoded at least in part by growth factor signaling that is mediated by sulfated residues of the extracellular matrix (ECM) in both axolotls and mice (Phan et al. 2015).

As envisioned by the PCM (French et al. 1976; Bryant et al. 1981), the positional information cells form a connective tissue grid that is arranged as a folded sheet within the dermis and surrounding all the internal tissues (e.g., muscle and bone) of the limb (Lheureux 1983; Gardiner & Bryant 1989; Holder 1989). The organization and continuity of positional information associated with this connective tissue grid has been mapped by grafting of internal skeletal tissues to difference positions around the limb circumference (Gardiner & Bryant 1989). As a consequence, no supernumerary structures form when the positional information (PI) of the graft and host tissues is the same. Conversely, ectopic limb structures (including entire limbs) are induced to form when the PI of graft and host tissues is different (Endo et al. 2004; Satoh et al. 2007).

The spatial distribution of this information grid has recently been demonstrated by the alternative approach of reprogramming the PI by retinoic acid (RA) (McCusker et al. 2014). RA treatment reprograms the PI of blastema cells to a value that corresponds to the posterior−ventral−proximal (PVPr) position in the limb grid (see Bryant & Gardiner 1992). By using the ALM (Fig. 6), ectopic blastemas can be induced at different positions around the limb circumference. When treated with exogenous RA, the blastema cells are reprogrammed to the PVPr identity and then interact with the surrounding cells of the PI grid to form supernumerary structures. In these experiments, reprogrammed anterior blastemas (induced to become PVPr by RA treatment) form multiple supernumerary limbs as a consequence of interacting with the surrounding anterior grid cells. In contrast, RA treated posterior blastemas do not form supernumerary limbs since the surrounding grid cells also have posterior PI.

## Origin of the positional information grid

**Figure 7:**
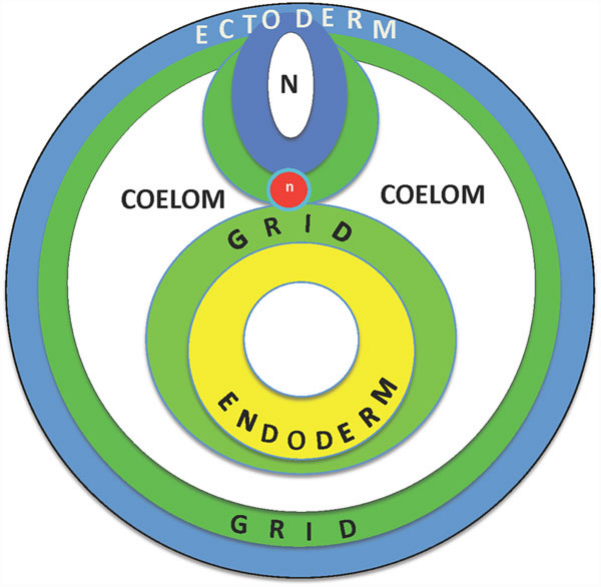
Origin of the positional information grid at gastrulation. A cross‐section through a stylized embryo after gastrulation, at which time the surface ectoderm (blue) is underlain by a layer of mesoderm (green), which also surrounds the developing neural tube and notochord. The endoderm of the gut tube is also surrounded by a layer of mesoderm, and the space between the gut‐associated and epidermis‐associated mesoderm is the coelom.

As discussed above, the PI grid most likely is a property of cells in the loose connective tissue, which historically have been referred to as fibroblasts. Given the lack of definitive cell markers, this is a relatively poorly characterized cell type in the body that arises very early in embryogenesis during gastrulation. At that time, cells of the future mesoderm migrate to form the mesenchymal layer of cells that lies between the epithelial layers of the ectoderm and endoderm. A cavity, the coelom, develops within the mesoderm, creating an outer mesodermal layer that underlies the ectoderm (the future dermis) and an inner layer that surrounds the endoderm (the future connective tissue surrounding the internal organs) (Fig. 7). Subsequent development of most organs occurs as a result of interactions between either ectoderm and mesoderm or endoderm and mesoderm. Studies of the development of vertebrate organs, based both on in vitro culture of organ anlage and in vivo experiments, have led to the conclusion that PI for the development of an organ is a property of the mesoderm layer (see Gilbert 2013). Studies of regenerating organs (especially the limb) have led to the same conclusion, as discussed in detail above. It thus appears that the PI grid needed for both the development and regeneration of organs and appendages is established in the early mesoderm around the time of gastrulation (Fig. 7). The implication of this view is that in those cases, such as in humans, where regeneration does not occur in response to injury, the challenge is to discover how to reactivate the inherent positional properties of the connective tissues that were used for pattern formation during embryonic development (Muneoka & Bryant 1982; Bryant & Muneoka 1986; Bryant et al. 2002).

Although most studies of the distribution of PI have involved regeneration of amphibian appendages, given the similarity of the conserved structural and developmental features of all parts of the body (arising through epithelial−mesenchymal interactions), we hypothesize that the PI grid provides the positional cues necessary both the development and regeneration of all organs. We have been fortunate that the surgical procedures needed to investigate the organization of positional information in amphibian appendages, including the tail and associated spinal cord, are compatible with survival and have led to regeneration outcomes that are expected as a result of intercalation of missing PI in the grid (French et al. 1976; Iten & Bryant 1976; Bryant et al. 1981). For other parts of the body, including most parts of the head and most internal organs, similar tests are not compatible with survival. When parts of these structures have been removed without affecting survival, they regenerate and replace the missing pattern (e.g., jaws, lens, and brain) (Ghosh et al. 1994; Suetsugu‐Maki et al. 2012; Maden et al. 2013).

Although regeneration of complex body parts in humans is limited to finger tips (see Muller et al. 1999; Han et al. 2005), there is evidence to conclude that we do in fact have a PI grid. Expression profiling of skin fibroblasts from different regions of the human body has demonstrated that there are position‐specific patterns of gene expression that are characteristic of different organs and appendages, as well as of different positions along appendages, such as the arm and leg (Chang et al. 2002). This global positional system encoded by gene expression patterns is probably the mammalian equivalent of the PI grid in salamander limbs. It is persistent and dynamically regulated by epigenetic modifications to chromatin (Rinn et al. 2006, 2008a, 2008b), as is suggested to be the case during salamander limb regeneration (McCusker & Gardiner 2014). This information system in humans presumably reflects the PI grid that functioned to generate body pattern during development, and is hypothesized to function to maintain this pattern during the homeostatic replacement of cells in the adult (Rinn et al. 2008a).

The role of the PI grid in mammalian regeneration cannot be tested directly; however, the regenerative response of axolotl wounds to the presence or absence of PI (the ALM, Endo et al. 2004) has been used to demonstrate that the ECM of skin from different regions of the mouse body has positional properties that influence regeneration. As with the response to skin grafts with different PI (discussed earlier), axolotl wounds also respond differently to grafts of cell‐free ECM grafts from different limb positions (Phan et al. 2015). Since these ECM grafts are decellularized, it is possible to assay for PI from mouse skin as well as from axolotl skin. Using this assay for PI, it is evident that mammalian ECM grafts can both induce and inhibit limb patterns in axolotl blastemas in a position‐specific, developmental‐stage‐specific, and heparan‐sulfate‐dependent manner (Phan et al. 2015). We thus hypothesize that the presence of a PI grid is a shared property of vertebrate development, and that the ability to access this information is essential for guiding regeneration responses leading to the replacement of lost structures.

## Control of pattern formation by gradients of diffusible morphogens

As discussed above, interactions between cells with PI regulate growth such that when cells with different PI communicate with each other growth is stimulated and when positional differences are resolved growth ceases. The next question is whether there is a reciprocal relationship such that growth controls gene expression leading to the establishment of different PI. Since intercalary growth leads to the replacement of the missing, intermediate PI when cells with different PI interact, it seems reasonable to consider that there is a mechanistically functional relationship between growth and pattern formation. Regardless of whether such a relationship exists, in the end new pattern (PI) arises via differential gene expression. The mechanisms controlling gene expression are well understood, and include both transcriptional (e.g., transcriptional activation and repression via transcription factors) and post‐transcriptional mechanisms (e.g., alternative splicing, miRNA and lncRNA). If growth can function to regulate PI, then the challenge is to identify how changes in growth lead to changes in gene expression.

Much of our understanding of mechanisms for regulating gene expression underlying pattern formation comes from studies of developmental genetics. These studies originated with the Nobel Prize winning studies of mutagenesis screens in Drosophila, in particular in the very early syncytial stage embryo where gradients of mRNA for key transcription factors are established within the oocyte cytoplasm as a result of the activities of the maternal follicle cells that surround the oocyte (see Gilbert 2013). The first mRNA gradient to be established is that of the transcription factor bicoid and, after fertilization, the bicoid mRNA is translated in the shared cytoplasm of the multinucleate, syncytial embryo to form a gradient of Bicoid protein (see Gilbert 2013). Nuclei exposed to different levels of the Bicoid protein established by intracytoplasmic diffusion then activate different genes leading to different cell fates after cellularization has occurred. Thus the Bicoid protein diffusion gradient is a direct demonstration that nuclei exposed to different levels of the same transcription factor can lead to the development of different cell fates (i.e., pattern formation). Although this transcription factor gradient arises by diffusion in the syncytial stage Drosophila embryo, it has remained unclear how gradients of transcription factors might be generated either at later stages of Drosophila development or in most other embryos, where the cells are mononucleate and transcription factors cannot diffuse freely.

**Figure 8:**
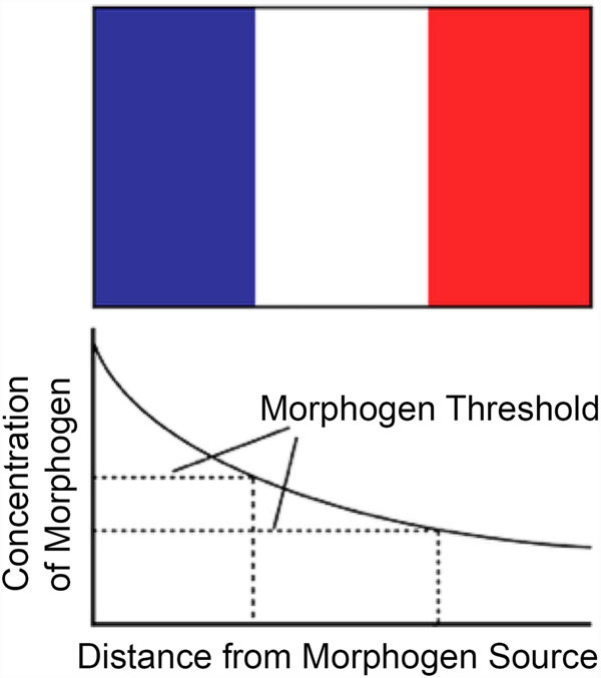
The French Flag model for positional information. Based on Wolpert (1969).

Unlike for the early Drosophila embryo, most studies of developing embryos have identified extracellular molecules (morphogens) as functioning to regulate differential gene expression, leading to pattern formation. This model is classically referred to as the French Flag model (Wolpert 1969), because it graphically illustrates how to divide a field of cells into three stripes of different cell fates or, for illustration purposes, into the red, white and blue stripes of the French flag (Fig. 8). Typically these extracellular patterning molecules are growth factor proteins and have dose‐dependent effects on cell fate. In developing embryos, morphogens are made at one end of a field of cells (signaling regions), and diffuse away from the source creating a gradient in which cells at one end are exposed to a high level of the extracellular morphogen and those at a distance from the source are exposed to low levels. This model is mechanistically rooted in the demonstration that different levels of a transcription factor (i.e., Bicoid protein) can regulate pattern formation across a field of cells. Thus the dominant view is that, within a developing field, cells respond to different concentration levels of an extracellular morphogen by activating morphogen‐dependent levels of expression of a specific intracellular transcription factor that in turn leads to pattern formation (Fig. 8). As discussed later, in many instances, morphogen sources are populations of cells that are either proliferating slowly or not at all, for example the floor plate of the neural tube and the zone of polarizing activity (ZPA) and the apical ectodermal ridge (AER) of the limb bud.

Aside from the data on the role of intracellular transcription factor gradients in pattern formation (i.e., Bicoid protein), the popularity of this model is largely a consequence of the elegance of the underlying mathematics of reaction−diffusion mechanisms (Turing 1952; Meinhardt 1982) that can generate and predict the emergence of biological patterns. By this view, pattern formation is a “reading out” of a chemical prepattern (Murray & Oster 1984). The challenge then is to identify explicit mechanisms for linking an extracellular graded morphogen (prepattern) with intracellular graded transcription factors (resulting in morphogenesis). Alternative models for pattern formation that are also based on elegant mathematics predict that pattern formation does not need to precede morphogenesis (i.e., no chemical prepattern), but rather that the mechanical morphogenetic properties of the cells and the ECM (e.g., migration, chemotaxis, contact guidance, and contact inhibition) lead to progressive pattern formation. By this view, pattern formation and morphogenesis “evolve simultaneously and in synchrony” (Murray & Oster 1984).

The latter view that pattern formation is an emergent property of cell−cell and cell−ECM interactions rather than a read‐out of chemical gradients is consistent with recent findings that morphogens may be distributed in gradients but that their function is not dependent on diffusion. For example, Wnts are evolutionarily conserved, diffusible morphogens; however, when experimentally engineered so as to be membrane‐tethered, they nevertheless are functional in controlling the pattern formation of Drosophila appendages (Alexandre et al. 2014). Similarly, the transforming growth factor β (TGF‐β) family member Decapentaplegic in Drosophila is a well‐characterized morphogen whose function is dependent on the presence of special filopodia referred to as cytonemes. These cell processes both send and receive morphogenetic signals resulting in cell−cell contact dependent function similar to what occurs in the nervous system (Roy et al. 2014). Finally, many of the canonical morphogens (e.g., FGFs and BMPs) are bound to the ECM via sulfated glycosaminoglycans (e.g., heparan sulfate proteoglycan, HSPG). In fact, their function as signaling molecules is dependent on being bound to the ECM, such that HSPGs are recognized as signaling co‐factors (Sarrazin et al. 2011). When grafted into regenerating axolotl wounds, cell‐free ECM from both axolotl and mouse skin influences pattern formation via an HSPG‐dependent mechanism (Phan et al. 2015).

These data indicating that diffusion is not necessary for morphogen function, coupled with data demonstrating that morphogen signaling can be mediated by short‐range cell−cell contact, suggest that it would be appropriate to revisit the issue of how morphogens control pattern formation. In particular, as discussed in detail above, given the strong evidence from the regeneration literature for a functional relationship between growth and pattern formation, we feel that it would be productive to examine critically the hypothesis that morphogens, many of which are known to affect cell division either positively or negatively, actually control pattern formation as a consequence of controlling growth. This reexamination of the functional mechanism of morphogens is timely in light of comments such as those by Lewis Wolpert himself: “Gradients of diffusible gradients are out. They are too messy to specify position reliably. I still believe in positional information, and the best evidence for it is intercalation” (Kerszberg & Wolpert 2007; Wolpert 2009). Our alternative view as to how morphogens control pattern formation has arisen historically from studies of regeneration that led to the PCM which, as discussed above, formalized the relationship between growth control and pattern formation. This functional relationship is the focus of the remainder of this paper.

## Control of pattern formation by growth

**Figure 9:**
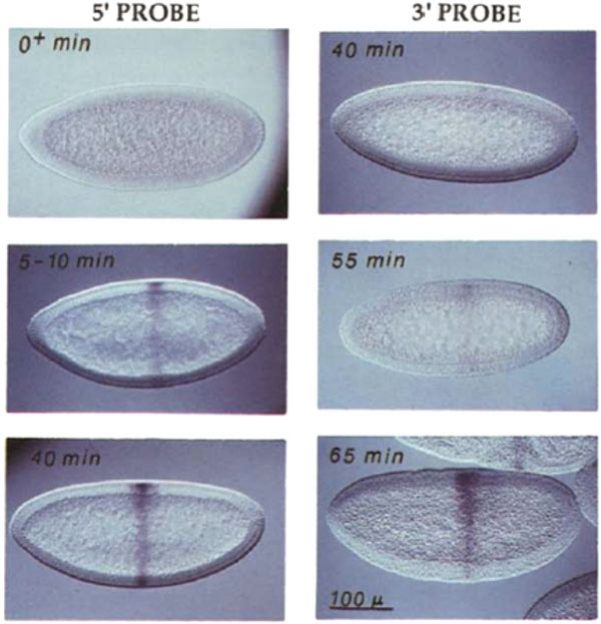
Expression of Ubx is gated by the cell cycle during the early stages of Drosophila embryogenesis. Initiation of transcription (5′ probe) is detected soon after fertilization (5−10 min), but completion of transcription of the entire transcription unit (detected by the 3′ probe) does not occur until cell cycle 14 when the length of the cell cycle increased (55 min). Thus the duration of the cell cycle during the first 13 mitotic divisions is too short to allow the completion of transcription of large genes. Modified from Shermoen and O'Farrell (1991).

Two reports in the literature inspired our early thinking about the mechanism by which growth could control pattern formation. The first study, based on the “intron delay hypothesis” proposed originally by Gubb (1986), demonstrated that the length of the cell cycle (G1 in particular) can gate transcription of genes (Shermoen & O'Farrell 1991). During the early cleavage stages of Drosophila embryos, the cell cycle is very short, of the order of 8 min during the first 13 mitotic cycles. Given the rate of RNA polymerization (variable, but on average approximately 1.4 kb/min), it takes about 55 min to transcribe the 77 kb Ultra‐bithorax (Ubx) transcription unit. Ubx transcripts first appear at cell cycles 14 and 15 when the duration of the cell cycle begins to increase and become asynchronous (Shermoen & O'Farrell 1991). Although the complete transcript of Ubx is not detected at earlier cell cycles, a probe to the 5′ region of the transcript detects expression as early as 5−10 min post‐fertilization (Fig. 9). In contrast, a 3′ probe does not detect Ubx expression until 55 min, at which point the cell cycle has increased to allow enough time for transcription of the entire transcription unit. For each of the early cell cycles, transcription halts and nascent transcripts are aborted as cells progress from G1 to S/M phase (Shermoen & O'Farrell 1991). Thus given a short G1 phase of the cell cycle relative to the size of the transcription unit for a gene, the cell cycle will function to regulate gene expression.

If the cell cycle can result in differential gene expression between populations of cells with different cell cycle kinetics, then regulation of the cell cycle (growth) could lead to the specification and differentiation of different cell fates (i.e., establish the fate map of the early embryo). This is in fact what appears to occur in the early Drosophila embryo (Foe 1989), which is the second report that directed our attention to the functional relationship between growth and pattern formation.

**Figure 10:**
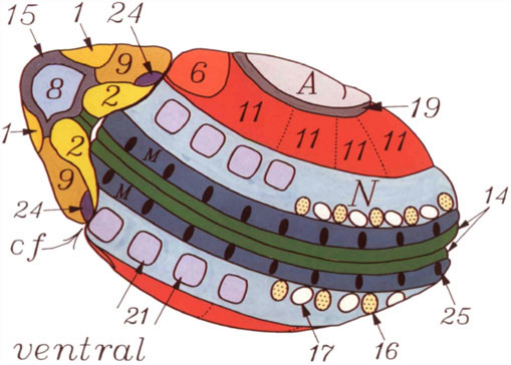
Mitotic domains correspond to the specification of cell fates. The cells within each numbered domain share the same cell cycle length and the same cell fate, both of which are different from adjacent domains with different cell cycle lengths and different fates. From Foe (1989).

When development of the Drosophila embryo (also typical of other embryos) is initiated at fertilization, the cell cycle characteristically lacks gap phases, is very rapid, and occurs synchronously within the blastomeres. No new mRNA is generated in this initial phase, and protein synthesis takes place using maternal mRNA synthesized and stored in the oocyte during oogenesis. As the cell cycle begins to lengthen, the transcription of the zygotic genome commences, which is a period in development referred to as the mid‐blastula transition (Edgar & O'Farrell 1989). As discussed above, in Drosophila embryos karyokinesis is globally synchronous among the nuclei of the syncytial embryos during the first 13 cell cycles. During cell cycle 14, the embryo undergoes cellularization, and domains of cell clusters arise that are locally synchronous in their cell cycle kinetics but are no longer in synchrony with cells in adjacent domains (Foe 1989). The timing and spatial distribution of these mitotic domains are reproducible between different embryos, and cells within a given domain share common behaviors and morphologies that are distinct from cells in adjacent domains (Fig. 10). Most importantly, the primordia of many of the larval structures arise from a specific mitotic domain (e.g., leg vs. wing).

The spatial and temporal correlation between cell cycle and developmental fate within a mitotic domain is evidence of a functional relationship in which growth can regulate pattern formation. Since the cells in mitotic domains are the progeny of early blastoderm cells, presumably there are different maternally derived cytoplasmic factors that are spatially localized and subsequently partitioned into each particular domain, and specify developmental fate by controlling the cell cycle kinetics. In a later section, we return to the question of the relationship between G1 length and cell fate, but based on these early experimental findings (Foe 1989; Shermoen & O'Farrell 1991) we hypothesize that the length of the cell cycle can alter expression of genes that influence the developmental fate of cells (Bryant et al. 1993; Ohsugi et al. 1997).

## Cell cycle times can be short and transcription units can be large: the role of G1 duration and intron size on the timing of gene expression

**Figure 11:**
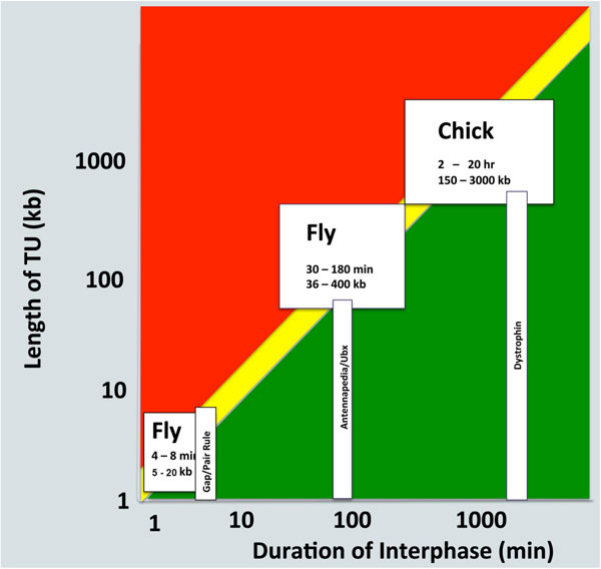
The relationship between transcription unit length and the length of interphase of the cell cycle. The vertical axis is transcription unit length, and the horizontal axis is the duration of interphase. Completion of transcription will occur in the green region, but transcripts will be aborted in the red region. The yellow zone indicates the situation in which transcription potentially could be gated.

The model of transcriptional gating by regulation of the cell cycle focuses attention on two parameters that determine the outcome in terms of pattern formation: (1) how are cell cycle kinetics regulated, and (2) how are changes in the size of transcription units regulated. As discussed above, the cell cycle can gate transcription if the size of the transcription unit is large relative to the length of G1 (Gubb 1986; Shermoen & O'Farrell 1991). This reciprocal relationship between gene length and the time window available for transcription is illustrated in Figure 11, which provides examples from two stages of development of Drosophila and from the chick embryo. This model is based on data indicating that the rate of eukaryotic transcription by RNA polymerase II is relatively uniform between cell types (see Ardehali & Lis 2009). For the data in Figure 11, we assumed a transcription rate between 1.2 and 2.4 kb/min and calculated the range of transcription unit lengths of genes that could or could not be transcribed over a given duration of interphase (illustrated as the yellow zone, Fig. 11). We note again that since RNA polymerization is restricted to the G1 phase of the cell cycle, and nascent transcripts are aborted when the cells progress beyond G1, the relevant window of time for transcriptional gating is the duration of G1 and not the total length of the cell cycle. Thus, for a given duration of G1, transcription units that are small enough will be able to complete transcription (green zone), but transcription will be aborted for transcription units that are too large (red zone).

Based on this model, it is evident that there are examples of genes in different organisms and at different times in development that are predicted to be regulated by cell cycle gating. We have already discussed these data for Ubx expression in Drosophila (Shermoen & O'Farrell 1991). During the early, syncytial stages of Drosophila development, the cell cycle is so short (8 min total, with an even shorter interphase) that only relatively small (less than 10 kb, Fig. 11) gap and pair rule genes can be completely transcribed in the time available. During embryogenesis, G1 lengthens at later stages of development, thus providing a longer window of time that would allow for the complete transcription of larger genes. Even with relatively longer durations of G1, such as reported for later stages of chick embryo development, there are genes that are too large to be transcribed (e.g., dystrophin).

Although an exhaustive search for genes with large transcription units is beyond the scope of this treatise, we note that there are a number of important developmental regulatory molecules with large transcription units, particularly in relationship to their much smaller mRNA that they are processed into. For example, the transcription factor Sox6 is transcribed from a DNA template that is nearly 600 kb long, yet has a protein coding region that corresponds to less than 3 kb of this sequence. Although more than 95.5% of the original RNA transcript is removed during mRNA processing, maintaining a relatively large transcription unit could be functionally important if the correct spatial and temporal expression of a gene is dependent on cell cycle gating of transcription.

**Figure 12:**
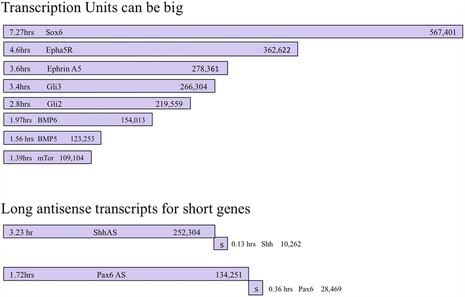
Some transcription (both sense and antisense) units for developmentally important genes can be very large.

Transcriptional gating by the cell cycle could occur either directly or indirectly. Our discussion to this point has focused on developmentally important genes that are too large to be transcribed when G1 is too short (direct gating). However, there are examples of very long antisense transcripts that would also be predicted to be gated by a short G1, and these overlap with important regulatory genes. Again, although we have not conducted an exhaustive search of the available databases, we note that the 5′ region of both shh and pax6 are each overlapped by the 3′ end of very large antisense transcripts (Fig. 12). Thus, although it may take only minutes to transcribe the target gene, it is predicted that it would take hours to completely transcribe the antisense transcript, which then would function indirectly to negatively regulate the function of shh or pax6. Thus gene expression and developmental fate can be altered directly or indirectly by shortening or lengthening the size of the gene, and by shortening or lengthening the duration of G1. Examples of both mechanisms are observed in nature, and thus presumably have been selected for during the course of evolution.

**Figure 13:**
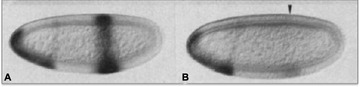
Variation in intron size between knirps and knirps‐related genes regulates timing of expression. The shorter version of the gene (Kni) is expressed at early stages of development when the cell cycle is short (left), but the longer version (Knrl) is not (right). Modified from Rothe et al. (1994).

Gene length can be altered by changing the size of introns present in the transcription unit without altering the coding region of the gene, which is the basis for the original intron delay hypothesis (Gubb 1986) as well as subsequent studies exploring the phenomenon of cell cycle gating of transcription (Shermoen & O'Farrell 1991; Thummel 1992; Rothe et al. 1994; Swinburne & Silver 2008; Takashima et al. 2011; Artieri & Fraser 2014). As is evident in Figure 11, if the length of G1 remains the same but the size of the transcription unit increases or decreases by changes in the size of introns, the transcription of that gene will be subject to cell cycle gating. In addition to either allowing for or preventing transcription at a given time in development, changes in the size of introns would also alter when and where in the embryo the gene product appears. Thus a decrease in intron size (shorter transcription unit) would cause a gene to be expressed earlier when G1 is shorter, or in a different population of cells (mitotic domain) with a shorter G1. A graphic example of the effect of variation in intron size is the comparative expression of the genes knirps (kni) and knirps‐related (Knrl) in Drosophila (González‐Gaitán et al. 1994; Rothe et al. 1994). These two genes have nearly identical coding regions (exons), but Knrl has three introns totaling 19 kb in addition to the 2 kb coding region. In contrast, the kni transcription unit is only 3 kb in total length. As a consequence, Kni is expressed at earlier stages with short cell cycle times that do not allow for transcription of Knrl, which begins to be expressed in post‐blastoderm stages where cell cycles are long enough for it to be made in the time available (Fig. 13). Both are thought to be derived from an ancestral gene with a large transcription unit, and the decrease in intron size of Kni is a consequence of selection by the rapid development (short G1) of the early embryo (Rothe et al. 1994). The hypothesis that intron size contributes to the timing mechanism of gene expression has also been tested experimentally (Takashima et al. 2011). Deletion of introns from the Hes7 locus involved in regulation of the somite segmentation clock in mice resulted in the loss of the oscillation of gene expression and severe segmentation defects.

**Figure 14:**
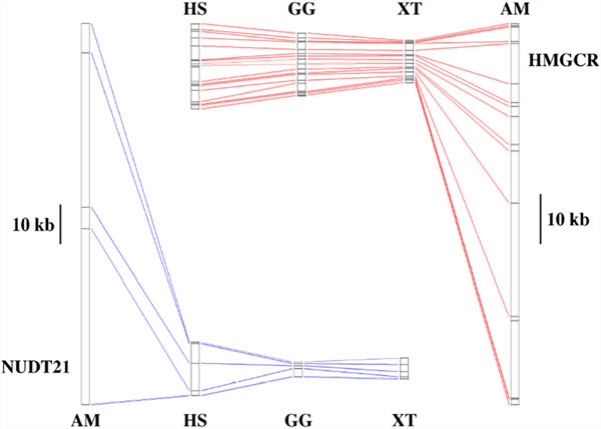
Axolotl genes have large introns. Comparison of intron lengths for two genes (NUDT21, cleavage and polyadenylation specific factor 5; HMGCR, 3‐hydroxy‐3‐methylglutaryl‐coenzyme A reductase) between the axolotl (AM), human (HS), chicken (GG), and frog *Xenopus tropicalis* (XT). From Smith et al. (2009).

Even more dramatic changes in the size of introns has occurred during vertebrate evolution, as evidenced by intron sizes in the axolotl, a urodele amphibian that is a model organism for vertebrate regeneration. The entire genome of a salamander species is yet to be sequenced and assembled, due at least in part to the large size of the genome, which at 30 Gb is about 10× the human genome. Although the entire axolotl genome has not been sequenced, several bacterial artificial chromosomes (BACs) have, and annotation of genes within those BACs shows that axolotl introns are on average 10× larger than orthologous vertebrate introns (Fig. 14; Smith et al. 2009). Although not extensively studied, cell cycle kinetics in these species presumably have become adjusted (slowed down) in order to compensate for the increased size of these exceptionally long transcription units. Thus with increasing size of transcription units as a consequence of increased intron size, the corresponding changes in the length of G1 of the cell cycle that would result in gating of transcription would occur on the time scale of hours rather than minutes as originally described in Drosophila. One question worth considering is whether gene lengthening and corresponding slower cell cycles may have conferred some selective advantages for salamanders, such as the ability to regenerate perfectly.

Finally, there is the question of how generalizable the intron delay hypothesis is to animals other than Drosophila. This is a question that has been raised repeatedly over the decades, and various authors have all concluded that the data from flies are likely to be broadly applicable (e.g., Thummel 1992). Specifically, there are data indicating that intron delay is occurring in early mammalian embryos (Graf et al. 2014). As discussed below, there are also data from a number of vertebrate models for discrete cellular domains with distinctive cell cycle kinetics (e.g., Boehm et al. 2010) that could be functionally equivalent to Drosophila mitotic domains (Foe 1989) in terms of gating transcription.

## Signaling regions, morphogens and growth factors

As noted above, gene expression and developmental fate are predicted to be altered by shortening or lengthening the duration of G1 relative to changes in the size of transcription units (Fig. 11). During the cell cycle, the durations of S phase (DNA synthesis leading to replication of the genome) and M phase (condensation of the genome and segregation of chromosomes to daughter cells) are less variable than G1 and G2 phases. Although G2 can be variable, and even absent in rapidly dividing cells, it is typically much shorter than G1, which is a period of high metabolic activity including transcription and translation of new gene products. In the absence of signals to progress to the S phase, cells can enter from an extended G1 phase to a more quiescent G0 phase, where they remain metabolically active and can be induced to progress in the cell cycle in response to extracellular signals (e.g., growth factors). Thus regulation of the duration of G1/G0 largely accounts for variation in the length of the total cell cycle. More important in the context of gene expression is that transcription is restricted to this period of the cell cycle, and nascent transcripts are aborted when the cells progress beyond G1. Thus the relevant window of time for transcriptional gating is the duration of G1, and extracellular signals that increase or decrease the length of G1 would be candidate factors for modulating cell cycle regulation of gene expression and developmental fate.

Over the past decades, a number of extracellular signaling factors have been isolated and characterized based on their ability to stimulate or inhibit cellular proliferation, and thus collectively are referred to as growth factors. More recently, studies of pattern formation have identified a number of these as having morphogenetic activities, leading to them being referred to as morphogens (e.g., FGF, BMP, TGF‐β, and WNT). Other signaling molecules that originally were identified as morphogens also function to control the cell cycle (e.g., RA and SHH). In recent years, the function of these signals as morphogens has attracted much research attention, whereas their function in cell cycle regulation has not. As discussed above, the dominant view of pattern formation is that cells respond to different concentration levels of an extracellular morphogen by activating expression of correspondingly different levels of a specific intracellular transcription factor that in turn leads to pattern formation (Fig. 8). The challenge for this view of pattern formation as a “reading out” of a morphogen gradient is to identify explicit mechanisms for linking the extracellular morphogen with the intracellular transcription factor. The model of cell cycle gated transcriptional regulation brings focus back to the functional role of morphogens as cell cycle regulators, and is based on a specific and testable mechanism by which morphogens, in their roles as growth factors (how they were originally discovered), also determine cell fate (based on more recent studies of pattern formation).

**Figure 15:**
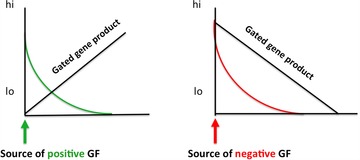
A model for how diffusion of extracellular growth factors would gate gene expression. High levels of a positive acting (green on the left) or a negative acting (red on the right) growth factor would establish a gradient of cell cycle times that in turn would establish a gradient of intracellular transcription factors.

We hypothesize that an extracellular growth factor/morphogen gradient links growth and pattern by shortening or lengthening the cell cycle in a graded, concentration‐dependent manner, thereby establishing an intracellular gradient of a cell cycle gated transcription factor across a field of cells. By this view, diffusion of an extracellular growth factor from a localized source would establish a gradient of cell cycle times from fast to slow in a dose‐dependent manner. Cells closest to the source of the signaling region will be maximally affected and those at a distance least affected, leading to a gradient of growth rates across a field of cells. Downstream of extracellular cell cycle control, expression of critical intracellular transcription factors will be gated (either directly or indirectly) in a corresponding gradient depending on whether the growth factor functions to stimulate or inhibit proliferation (Fig. 15).

Both growth stimulating (positive acting) and growth inhibiting (negative acting) factors can lead to the establishment of an intracellular transcription factor gradient (Fig. 15). In response to a gradient of a cell cycle shortening growth factor/morphogen (e.g., FGF or WNT), cells nearest to the source will have a short cell cycle and be unable to transcribe long genes (green, Fig. 15). At positions that are progressively further away from such a morphogen source, the length of G1 will increase and a long gene product will begin to accumulate. Conversely, in response to a gradient of a cell cycle lengthening morphogen (TGF‐β or RA), cells near to the source will have a long cell cycle and be able to transcribe long genes (red, Fig. 15). At a distance from such a morphogen source, G1 will be shorter, and less of a long gene product will be transcribed. In both cases, the cells at one end of the gradient will have a long cell cycle and those at the other will have a short cell cycle.

**Figure 16:**
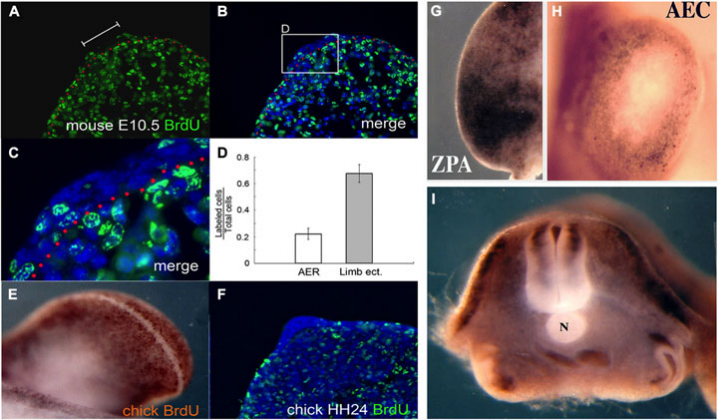
Growth factor and morphogen signaling regions are associated with withdrawal from the cell cycle and non‐proliferation. Low or no proliferation of cells in these regions are observed in the mouse AER (A−D), the chick AER (E, F), the chick ZPA (G), the axolotl AEC (H), and the chick notochord (I). BrdU immunohistochemistry in either sectioned tissues (A−C, F) or in whole‐mount preparations (E, G−I). Modified from Ohsugi et al. (1997) and Satoh et al. (2012).

The localized population of cells that functions as the source of the growth factor/morphogen is classically referred to a signaling center (e.g., the ZPA of developing limb buds). We consider it noteworthy that many of these classical signaling centers themselves exhibit unique cell cycle kinetics, specifically a very long G1/G0 phase (Fig. 16; Hay & Fischman 1961; Ohsugi et al. 1997; Satoh et al. 2012). These regions include the AER of both mouse (Fig. 16A−D) and chick (Fig. 16E, F) limb buds, the ZPA of the chick limb bud (Fig. 16G), the apical epithelial cap (AEC) of regenerating axolotl limbs (Fig. 16H), the notochord and floor plate of the chick embryo (Fig. 16I), the AEC of regenerating lizard tails and zebrafish fin rays, the enamel knot of developing teeth, and the midbrain−hindbrain boundary (Cox 1969; Bellomo et al. 1996; Ding et al. 1998; Jernvall et al. 1998; Poleo et al. 2001; Nechiporuk et al. 2003; Poss et al. 2003; Trokovic et al. 2005). Among these, there is experimental evidence that the signaling function of the AEC is dependent on the signaling cells having a long cell cycle (Satoh et al. 2012). The AEC is required for blastema formation and outgrowth during salamander limb regeneration (Thornton 1960), and this function in turn is dependent on signaling from nerves (Singer 1952, 1974; Endo et al. 2004; Satoh et al. 2012). Withdrawal of basal keratinocytes of the AEC from the cell cycle is one of the earliest events in regeneration (Hay & Fischman 1961; Satoh et al. 2012). This coordinated regulation of cell cycle kinetics is associated with the presence of gap junctions between the cells of the AEC and is dependent on signaling by nerves. In the absence of nerve signals, AEC basal keratinocytes enter the cell cycle, are highly proliferative, and regeneration fails (Satoh et al. 2012).

Historically, analyses of endogenous cell kinetics in both embryonic and regenerating tissues have assumed that the cells are dividing asynchronously with similar cell cycle kinetics, which in fact they are not (e.g., Boehm et al. 2010; see discussion above about populations of cells with a very long G1/G0 phase). This is particularly a problem for proliferation studies of blastema cells of regeneration limbs since they are a highly heterogeneous population of cells (Muneoka et al. 1986; Kragl et al. 2009; McCusker & Gardiner 2013). In more recent times, techniques for higher resolution analysis of cell cycle kinetics have been developed and applied to the developing mouse limb bud (Boehm et al. 2010). The results of this study identified discrete domains of cells with different cell cycle kinetics, that is, areas of low proliferation (proximal−central) and others of high proliferation (dorsal and ventral as well as anterior−distal). Thus moving forward to better understanding the co‐regulation of the cell cycle and gene expression will necessitate utilizing techniques for high temporal and spatial resolution of cell cycle kinetics (e.g., Boehm et al. 2010).

In addition to signaling centers that arise endogenously during development and regeneration, ectopic signaling centers can be induced experimentally. Based on the distribution of RA in developing embryos (Eichele & Thaller 1987; Thaller & Eichele 1987) and regenerating limbs (Monaghan & Maden 2012), endogenous RA is associated with the establishment of signaling regions (e.g., ZPA in limb buds, ventral region of the neural tube, and AEC of blastemas). From a number of experimental studies based on treating developing embryos and regenerating salamander limbs with RA, the positional identity of the cells is reprogrammed by RA to form ectopic signaling centers (e.g., ZPA, Noji et al. 1991; Wanek et al. 1991; Bryant & Gardiner 1992). The ability to be reprogrammed is dependent on the state of differentiation of the cells such that undifferentiated cells in the embryo are responsive to RA, whereas during regeneration undifferentiated blastema cells are reprogrammed but uninjured (differentiated) limb cells are not (Maden 1983; McCusker & Gardiner 2014; McCusker et al. 2014).

Reprogramming of positional information by RA has been directly demonstrated by its ability, or lack of ability, to induce ectopic limbs at different positions around the limb circumference (McCusker et al. 2014). RA treatment of blastemas in anterior and dorsal locations, but not posterior and ventral locations, results in the induction of complete ectopic limbs. Cells with different positional information interact via intercalation to stimulate growth and pattern formation (as discussed above), and thus position‐specific differences in limb induction indicate that there are differences in the positional disparity between the RA‐reprogrammed blastema cells and the non‐reprogrammed cells at the periphery of the wound. From these and other observations, RA reprograms the positional information of blastema cells to a posterior−ventral (also proximal) position on the limb, since supernumerary limbs are induced at those positions. Importantly, exposure of blastema cells to RA inhibited proliferation during the period of reprogramming (Maden 1983), a phenomenon that now appears relevant in the context of the correlation between cell cycle kinetics and pattern formation.

**Figure 17:**
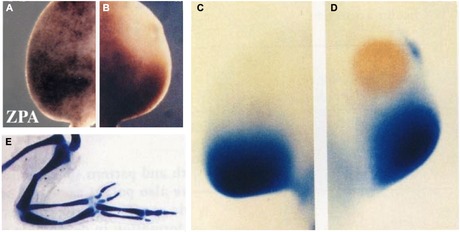
Experimentally changing cell cycle kinetics changes the pattern. Cells of the ZPA typically do not incorporate high levels of BrdU, in contrast to adjacent cells in more anterior positions (A). Localized delivery of amphidicolin by a microcarrier bead blocks the cell cycle and prevents incorporation of BrdU (B). In response, expression of the ZPA marker gene BMP2 (C) is induced ectopically in the anterior (D) and an extra digit is induced (E). Whole‐mount anti‐BrdU staining (A, B); BMP2 in situ hybridization (C, D); and whole mount skeletal staining (E). Modified from Ohsugi et al. (1997).

Finally, experimentally inducing cells in the chick wing bud to withdraw from the cell cycle induces them to acquire ZPA‐like signaling (Ohsugi et al. 1997). Cells within the ZPA, like those in many other signaling centers, have long cell cycles (Figs 16, 17A). Cells outside of the ZPA can be induced to withdraw from the cell cycle by localized treatment with aphidicolin, an agent used to synchronize cells in the cell cycle by reversibly inhibiting DNA replication (S phase). In response to an implanted bead soaked in aphidicolin, cells in the anterior region of the limb bud withdraw from the cell cycle and no longer incorporate BrdU (Fig. 17B). Coincident with cell cycle withdrawal, a posterior/ZPA marker gene, Bmp2 (Fig. 17C), is expressed ectopically in the anterior region (Fig. 17D). In addition, a supernumerary digit is induced to form on the anterior of the limb (Fig. 17E), which is comparable to the response observed when either the ZPA or an RA bead is grafted into the anterior. Thus both RA and aphidicolin extend the length of G1 and induce ectopic pattern formation. The endogenous function of RA as a signaling molecule may also be a consequence of altering the length of the cell cycle leading to establishment of signaling centers in each successive field of cells as development progresses.

## Implications of a functional link between growth and pattern formation

There are a number of ways in which a GRN could evolve so as to be sensitive to cell cycle gating. Although the network could be very simple such that a target transcription factor is directly gated by the cell cycle because it is derived from a relatively large transcription unit, it could also be complex so long as at least one of the regulators in the network has a large enough transcription unit to be gated. Regardless of whether the critical transcription factor is directly or indirectly gated, any large transcript (either coding or non‐coding) has the potential to be cell cycle gated and thus may function as a regulatory factor in a GRN, particularly in a developing embryo with short cell cycle times.

**Figure 18:**
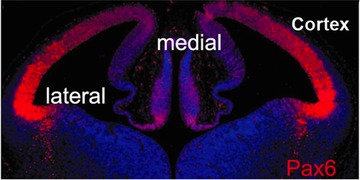
Graded distribution of a transcription factor. Pax6 expression in the E12.5 developing mouse cortex. Modified from Manuel et al. (2015).

Based on the observed relationship between growth and pattern formation in regeneration (French et al. 1976; Bryant et al. 1981), it is likely that genes involved in pattern formation are regulated either directly or indirectly by GRNs in which at least one of the regulators is transcribed from a large transcription unit. Similarly, developmentally important genes that exhibit a graded pattern of expression in development (e.g., Pax6; Fig. 18; Manuel et al. 2015) are predicted to be part of a GRN that is regulated by a large transcription unit. Thus special attention should be paid to large transcription units and/or transcription factors that exhibit a graded pattern of gene expression.

Transcriptional units that do not code for a gene product can also be targets for cell cycle gating, and in turn function to regulate expression of genes that encode proteins. The complex functions of non‐coding RNAs are now well understood and appreciated, and with advances in genomics we anticipate the discovery of long non‐coding transcription units that are potentially cell cycle gated. In particular, long non‐coding RNA with reverse‐complement sequence to developmental regulatory genes (e.g., shh and pax6; Fig. 12) would be likely candidates for cell cycle gated regulatory factors.

It is also possible that some morphogens become expressed as an extracellular gradient because they are themselves regulated by cell cycle gating of transcription (either directly or indirectly) rather than because they diffused from a localized source. This presents somewhat of a “chicken and egg” problem in terms of how a cell cycle regulator can be regulated by the cell cycle. However, since the end result (an intracellular gradient of a transcription factor) results from a gradient of cell cycle times (short in one region and long in another), any developmental event (morphogen induced or not) that alters cell cycle kinetics could initiate a cascade of downstream events leading to transcriptional gating. Thus in the case of a gated morphogen, the initial change in cell cycle length would lead to creation of a population of cells expressing a higher or lower level of morphogen. As cells divide and move away from the initial population of signaling cells, their cell cycle duration changes, which in turn leads to a change in the level of expression of the gated morphogen. Regardless of the details of such GRNs, our model predicts that agents that change the length of the cell cycle change the expression of genes that can be gated either directly or indirectly leading to pattern formation.

The model of cell cycle gating has implications for evolution since both cell cycle kinetics and transcription unit length are subject to variation upon which natural selection can operate. Agents that shorten/lengthen the cell cycle or shorten/lengthen genes can alter the timing of expression of genes that influence the form and function of the embryo. As Stephen Jay Gould pointed out, changes in the relative timing at which characters appear in development creates the heterochrony upon which natural selection can operate (Gould 1977).

Beyond development and regeneration from which we derived the data underlying the model of cell cycle gating, we also note that dysregulation of the cell cycle is a hallmark of cancer. This raises the question of whether cell cycle regulation is upstream or downstream of differentiation. Typically proliferation of tumor cells is written about as a downstream consequence of carcinogenesis. Similarly, differentiation and dedifferentiation seem to be conceptualized as being upstream of proliferation. In contrast, the cell cycle gating model places proliferation upstream of gene expression that regulates the state of differentiation. Revisiting the cause and effect relationship between proliferation and differentiation could provide insights into regulating the state of differentiation of tumor cells.

In conclusion, our proposed model of cell cycle gated transcriptional regulation brings focus back to the functional role of morphogens as cell cycle regulators, and proposes a specific and testable mechanism by which morphogens, in their roles as growth factors (how they were originally discovered), determine cell fate (based on more recent studies of pattern formation). Our goal in writing this treatise is to challenge the scientific community to use the power of strong inference (Platt 1964) to consider alternative hypotheses for the mechanisms of pattern formation. In so doing, there is the opportunity to use modern methodologies and the power of genetics and genomics to test the predictions they make. Since our views have arisen from studies of regeneration for which the functional link between growth and pattern formation is strikingly evident, we would also consider the possibility of a function link between large genome size, long cell cycles, and regeneration. At the least, animals such as the axolotl with legendary regenerative abilities, coupled with a very large genome rich in sequences for long transcripts, will emerge as useful models in which to explore the relationship between gene size and cell cycle in development, pattern formation, and evolution.

## Financial Disclosure

Research was funded in part by a Defense Advanced Research Projects Agency (DARPA) subcontract from Tulane University (TUL 519‐05/06), a US Army Multidisciplinary University Research Initiative (MURI) subcontract from Tulane University (TUL 589‐09/10), and the National Science Foundation through its support of the Ambystoma Genetic Stock Center at the University of Kentucky, Lexington. The funders had no role in study design, data collection and analysis, decision to publish, or preparation of the manuscript.
